# Supplementary material for: Causal Relationship between Aging and Anorexia Nervosa: A White-Matter-Microstructure-Mediated Mendelian Randomization Analysis
Source: Biomedicines. 2024 Aug 16;12(8):1874. doi: 10.3390/biomedicines12081874 (PMC11351342; doi:10.3390/biomedicines12081874)
Supplement: Supplementary file 1 [file biomedicines-12-01874-s001.zip › Supplementary Figure/Figure S4/Singe SNP plot of MR analysis for TL to ubm-b-2015.pdf]

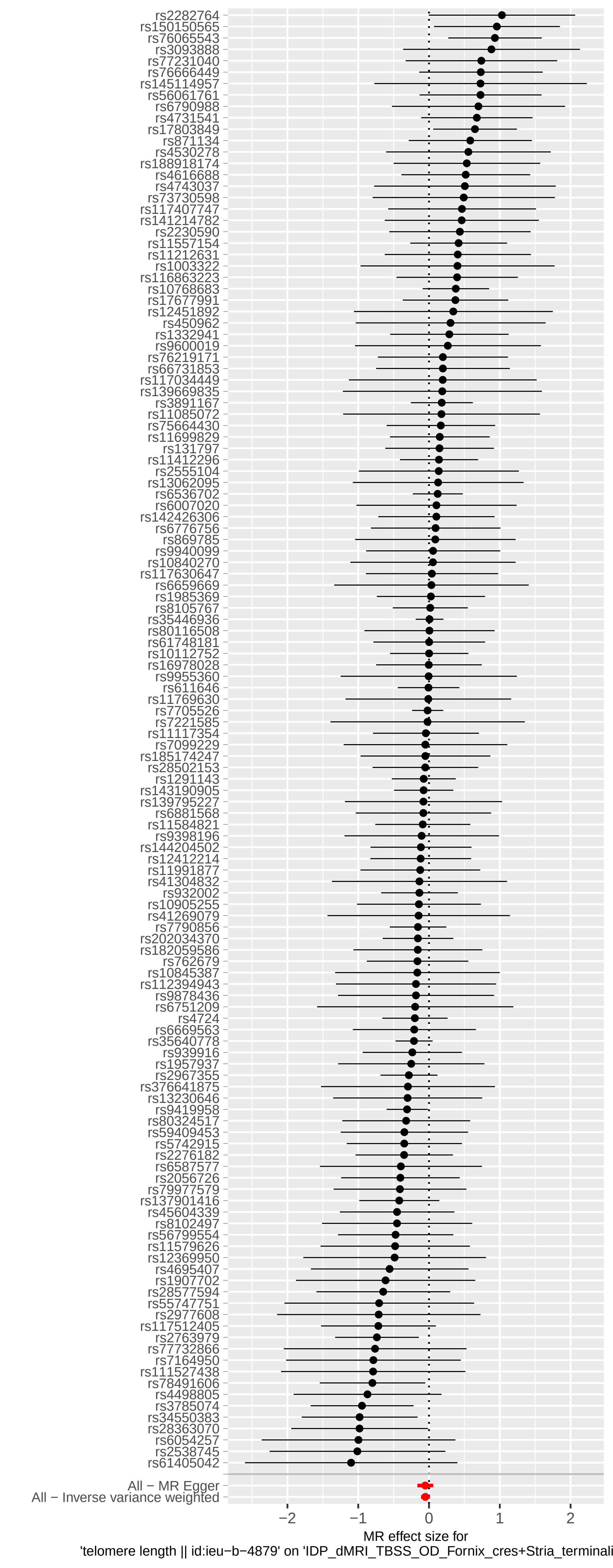
rs2282764
rs150150565
rs76065543
rs3093888
rs77231040
rs76666449
rs145114957
rs56061761
rs6790988
rs4731541
rs17803849
rs871134
rs4530278
rs188918174
rs4616688
rs4743037
rs73730598
rs117407747
rs141214782
rs2230590
rs11557154
rs11212631
rs1003322
rs116863223
rs10768683
rs17677991
rs12451892
rs450962
rs1332941
rs9600019
rs76219171
rs66731853
rs117034449
rs139669835
rs3891167
rs11085072
rs75664430
rs11699829
rs131797
rs11412296
rs2555104
rs13062095
rs6536702
rs6007020
rs142426306
rs6776756
rs869785
rs9940099
rs10840270
rs117630647
rs6659669
rs1985369
rs8105767
rs35446936
rs80116508
rs61748181
rs10112752
rs16978028
rs9955360
rs611646
rs11769630
rs7705526
rs7221585
rs11117354
rs7099229
rs185174247
rs28502153
rs1291143
rs143190905
rs139795227
rs6881568
rs11584821
rs9398196
rs144204502
rs12412214
rs11991877
rs41304832
rs932002
rs10905255
rs41269079
rs7790856
rs202034370
rs182059586
rs762679
rs10845387
rs112394943
rs9878436
rs6751209
rs4724
rs6669563
rs35640778
rs939916
rs1957937
rs2967355
rs376641875
rs13230646
rs9419958
rs80324517
rs59409453
rs5742915
rs2276182
rs6587577
rs2056726
rs79977579
rs137901416
rs45604339
rs8102497
rs56799554
rs11579626
rs12369950
rs4695407
rs1907702
rs28577594
rs55747751
rs2977608
rs117512405
rs2763979
rs77732866
rs7164950
rs111527438
rs78491606
rs4498805
rs3785074
rs34550383
rs28363070
rs6054257
rs2538745
rs61405042
All − MR Egger
All − Inverse variance weighted
−2
−1
0
1
2
MR effect size for
'telomere length || id:ieu−b−4879' on 'IDP_dMRI_TBSS_OD_Fornix_cres+Stria_terminali
